# Organophosphate poisoning at Chris Hani Baragwanath Academic Hospital 2012 - 2015

**DOI:** 10.7196/AJTCCM.2019.v25i3.001

**Published:** 2019-09-17

**Authors:** J Bruins, C N Menezes, M L Wong

**Affiliations:** 1 Division of General Medicine, Department of Internal Medicine, Chris Hani Baragwanath Academic Hospital and Faculty of Health Sciences, University of the Witwatersrand, Johannesburg, South Africa; 2 Division of Infectious Diseases, Department of Internal Medicine, Chris Hani Baragwanath Academic Hospital and Faculty of Health Sciences, University of the Witwatersrand, Johannesburg, South Africa; 3 Division of Pulmonology, Department of Internal Medicine, Chris Hani Baragwanath Academic Hospital and Faculty of Health Sciences, University of the Witwatersrand, Johannesburg, South Africa

**Keywords:** organophosphate, poisoning, South Africa

## Abstract

**Background:**

Patients with acute organophosphate poisoning are frequently admitted to the Chris Hani Baragwanath Academic Hospital (CHBAH), and yet there is little literature assessing aspects of these admissions.

**Objectives:**

To determine the demographic profile, common clinical and biochemical findings, use of prognostic tools (APACHE II), management and outcome of adult patients admitted to the high care area (HCA) and intensive care unit (ICU) at CHBAH.

**Methods:**

A retrospective data analysis of hospital records for 129 patients admitted to the HCA and ICU at CHBAH for the period 2012 - 2015 was undertaken. The demographic profiles and clinical and biochemical presentations of the patients were determined, together with their subsequent management and outcomes. Use of the APACHE II score as a prognostic tool was evaluated, and the average enzyme inhibition levels demonstrated by the patients was assessed.

**Results:**

The median age of the group was 30 years, with 68.2% being male. The most common clinical finding was pinpoint pupils (96.1%) followed by a Glasgow Coma Score <13 (85.3%), fasciculations (60.5%), diarrhoea (37.2%) and seizures (10.1%). Admissions to the HCA (52.7%) predominated, with the majority of patients requiring ventilator support (99.2%). The mean (SD) duration of stay was 6.8 days for ICU (6.4) and 3.7 days for HCA (5.2). The overall mortality rate was 5.4%. Standard treatment was intravenous atropine. Blood results reflected low levels of acetylcholinesterase enzyme activity. The APACHE II score was underutilised.

**Conclusion:**

The findings of the study underscore the frequent use of organophosphate compounds in our area. Further studies across the country will help to highlight the magnitude of the consequences of organophosphate poisoning, as well as the burden imposed on limited healthcare resources.

## Background

Since the first organophosphate that inhibited acetylcholinesterase activity was synthesised in 1854, the development of organophosphate compounds has progressed rapidly; most are used in the agricultural industry, although their uses extend to the plastics and oil industries, as well as the pharmaceutical industry.^[1]^

Acetylcholinesterase inhibitors include the pure organophosphates, which irreversibly inhibit the enzyme acetylcholinesterase, resulting in excess acetylcholine at the synapse, with the return of enzyme function entirely dependent on the synthesis of new enzyme units. Another class includes the carbamates, which are chemically similar to the pure organophosphates but result in reversible acetylcholinesterase inhibition as the bonds between the poisonous compound and the acetylcholinesterase enzyme break down spontaneously and the enzyme regains its function in about 48 hours.^[2]^ Although these differences exist, both classes of toxin, once ingested, present with the clinical features of the cholinergic toxidrome which, together with an exposure history, provides the basis for the diagnosis of organophosphate or carbamate poisoning in most settings.^[3]^

There has been increasing interest in acetylcholinesterase inhibitors internationally, owing to their potential for use in acts of bioterrorism; but, in developing countries such as our own, the emphasis is still on exposure in the spheres of domestic and farming applications, as well as their use for self-harm, all of which have been noted to be on the rise in recent years.^[3]^ This rise has been supported by increasing accessibility of these agents, which are often sold illegally as more cost-effective options than other commercially available pesticides in urban areas.^[4]^

Owing to their acute toxicity, significant exposure to these agents can result in severe clinical effects in patients, requiring not only hospital admission, but possibly also intensive care level intervention. Intensive care services are under immense strain from lack of resources. Patients suffering from intentional poisoning add to the already significant burden of trauma and disease.

In our study, we have used the term organophosphate poisoning to encompass the cholinergic syndrome produced by both pure organophosphates as well as by carbamate poisoning. This is because of both its retrospective nature, with an inability to access either blood samples or the actual poison samples from that period, and also the fact that in our resource-constrained setting, access to the necessary laboratory tests to differentiate between the two is limited.

Although organophosphates, a term which as indicated includes carbamates in the context of our article, appear to be freely available in South Africa (SA), the health impact of the subsequent poisonings, as demonstrated in other developing nations, has far-reaching consequences for the patients, their families and communities, and the local health system as a whole.[5,6] Unfortunately, few studies have been conducted on the topic of organophosphate poisoning in our region in recent years.

## Objectives

The objectives of this study were to determine the demographic spectrum of patients admitted to the medical high care unit (HCA) and intensive care unit (ICU) with organophosphate poisoning, and to illustrate the clinical profiles of these patients on admission. We also assessed whether the APACHE II score had been calculated, in order to determine its prognostic relevance in our setting. Further objectives included determining the average length of stay requirement for ventilation, and duration thereof, for these patients as well as the associated mortality rate.

## Methods

### Population

A retrospective review of the files of all patients over 18 years of age, admitted with a diagnosis of organophosphate poisoning to the HCA and the ICU of Chris Hani Baragwanath Academic Hospital (CHBAH) from 1 January 2012 to 31 December 2015, was conducted. Participants in the study were those with either a clinical diagnosis of organophosphate poisoning, positive history of ingestion and/or an appropriate clinical response to atropine.

### Data collection

Details of patients admitted to each ward with organophosphate poisoning were obtained from the ward admission register. Using these details, patient files were obtained from the records department, and their demographic profile, including gender, ethnicity, age and reason for ingestion, was extracted. Whether the patients presented with any of the following clinical signs on admission was then noted: pupil size (pinpoint or not), salivation, fasciculations, seizures, diarrhoea and Glasgow Coma Scale (GCS) score <13, as well as their vital signs, including blood pressure, pulse and respiratory rate. The results of their initial arterial blood gas analysis were noted, as well as blood activity levels of red cell cholinesterase or pseudocholinesterase, if measured. It was then noted to which ward (HCA/ICU) they were admitted, whether they required ventilation and, if so, the duration thereof, as well as their length of ward stay and outcome. Values for the APACHE II score were noted if calculated. This information was then entered into data collection sheets.

### Statistical analysis

The information was entered into Excel spreadsheets and then further analysed using SAS (SAS, USA). Descriptive analysis of the data was carried out with categorical variables being summarised by frequency and percentage tabulation, and illustrated by means of histograms. Continuous variables were summarised using means and standard deviations or medians and interquartile ranges, and their distribution was illustrated by means of histograms.

Fisher’s exact test was used to assess the relationship between the two wards and their associated mortality, ventilation and outcome. Strength of the associations was measured by the phi coefficient. The relationship between each ward and duration of ventilation and length of stay was assessed by the Wilcoxon rank sum test. Strength of the associations was measured by the r-value. A 5% significance level was used.

## Results

A total of 159 adults were admitted to the HCA and ICU at CHBAH over the study period. Of this number, 129 data sets were included in the study (81.1%), owing to incomplete files, or files with substantial missing data, for 30 patients.

**Fig. 1:**
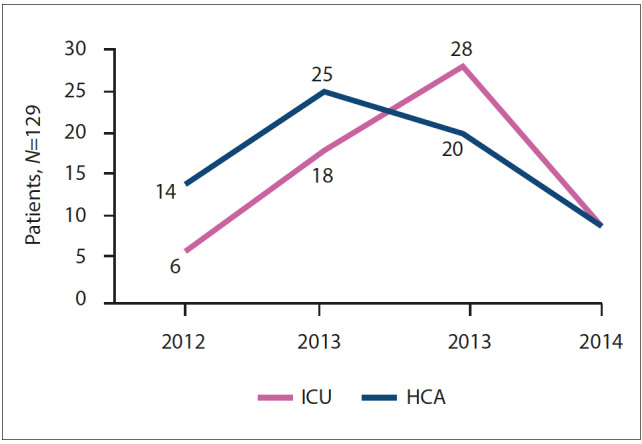
Yearly OPP admissions to Chris Hani Baragwanath Academic Hospital OPP = organophosphate poisoning ICU = intensive care unit HCA = high care area

Our study showed a rapid rise in the number of patients admitted to the HCA/ICU with organophosphate poisoning from 2012 to 2014, but a decline in 2014 (Fig. 1).

### Demographic profile

In keeping with the area demographic profile, the majority of patients were black (99.2%). The median age of the patients was 30 years, with the overall majority of them being male. The reason for ingestion in the study population was attempted suicide in 85.1% of the patients, with alleged homicide and accidental ingestion comprising the rest. No reason for ingestion was documented in 15 cases (11.6%) (Table 1).

Unfortunately, in only 2 files was the source of the poison documented (street vendor), which could not be statistically interpreted.

**TABLE 1:** Demographic profile, clinical and biochemical characteristics of patients admitted to HCA/ICU at Chris Hani Baragwanath Academic Hospital with OPP (N=129)

Variable	*n*(%)*
Age (median)	30
Gender	
Male	88 (68.2)
Female	41 (31.8)
Ethnicity	
Black	128 (99.2)
Indian	1 (0.8)
Reason for ingestion	
Suicide	97 (75.2)
Alleged homicide	9 (7.0)
Accidental	8 (6.2)
Not stated	15 (11.6)

HCA = high care area

ICU = intensive care unit

OPP = organophosphate poisoning

* Unless otherwise specified

### Clinical and biochemical presentation

**Fig. 2:**
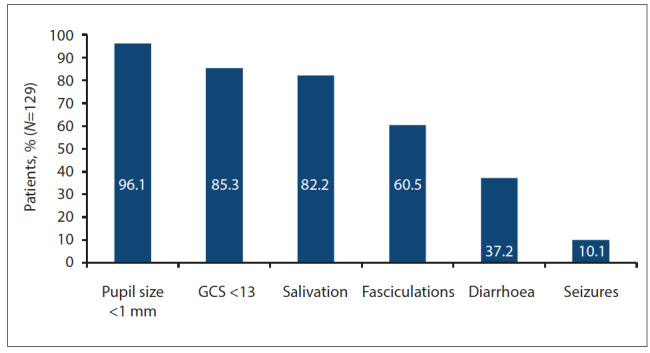
Frequency of clinical signs of OPP patients on admission OPP = organophosphate poisoning GCS = Glasgow Coma Scale

The most common clinical sign documented on presentation of the patients was pinpoint pupils (<1 mm), which was present in 96.1%. Thereafter, a GCS score <13, salivation, fasciculations, diarrhoea and seizures were noted in descending order of frequency (Fig. 2).

The mean (SD) systolic blood pressure was 132 (25) mmHg, the diastolic BP 80 (18) mmHg, pulse rate 85 (28) beats per minute (bpm) and the respiratory rate was 18 (5) breaths per minute (Table 2). Evaluation of arterial blood gases showed that the mean (SD) arterial pH value was 7.16 (0.15), with a predominant respiratory acidosis (Table 2).

**Table:** Clinical and biochemical characteristics of patients admitted to HCA/ICU at Chris Hani Baragwanath Academic Hospital with OPP

Characteristics	Mean (SD)*
BP (systolic)	132 (25)
BP (diastolic)	80 (18)
Pulse	85 (28)
Respiratory rate	18.1 (4.8)
RCC (median (IQR))^†^	400 (355 - 766)
PCHE (median (IQR))^†^	200 (200 - 704)
APACHE II score^†^	12
pH (median)	7.16
PaO_2_ (median)	79
PaCO_2_ (median)	52
BE (median)	–10.1
HCO_3_ (median)	19.0

HCA = high care area; ICU = intensive care unit

OPP = organophosphate poisoning; BP = blood pressure

RCC = red cell cholinesterase; IQR = interquartile range

PCHE = pseudocholinesterase; APACHE = applied physiology and chronic health evaluation score

PaO_2_ = partial pressure of oxygen; PaCO_2_ = partial pressure of carbon dioxide

BE = base excess; HCO_3_ = bicarbonate

* Unless otherwise specified

^†^ All scores calculated with a total of 129 patients except for RCC (n=20), PCHE enzyme activity levels (n=86) and APACHE scores (n=13)

Red cell cholinesterase was measured in only 25 cases, with results available for only 20 cases. The median value was 400 U/L (IQR 355 - 766; range 23 - 6 232) (laboratory reference range 4 752 - 8 225 U/L). Pseudocholinesterase levels were measured in 87 cases, with 86 results being available. The median value was 200 U/L (IQR 200 - 704; range 200 - 6 039) (laboratory reference range 4 620 - 11 500 U/L for males and 3 930 - 10 800 U/L for females) (Table 2).

### Treatment

The patients included in this study were treated with intravenous atropine infusions only. There were no instances where any oxime or any other agent was used.

### Mechanical ventilation

Of the patients included in the study, 99.2% required ventilation. Only one patient, who survived, did not receive ventilatory support.

**Fig. 3:**
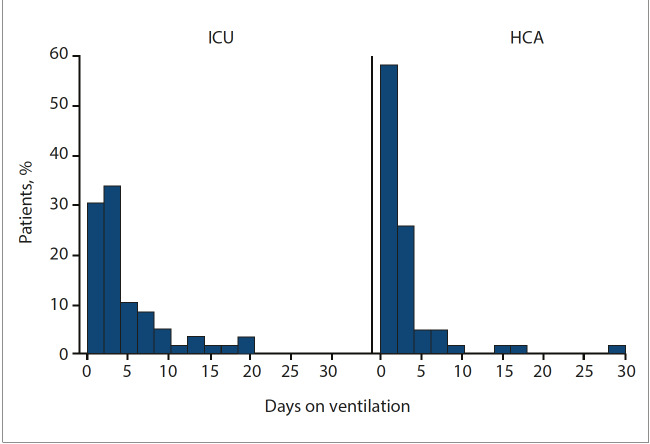
Ward comparison of days on ventilation. ICU = intensive care unit HCA = high care area

When analysing the number of ventilator days, the seven patients who died were excluded from the analysis. Of interest, two of the patients who died had prolonged durations of ventilatory support (22 and 24 days, respectively), whereas the remainder had durations that fell into the IQR demonstrated by the survivors. Of the 121 survivors who required ventilatory support, the median duration of ventilatory support was longer for ICU patients (two days) than for HCA patients (one day) (Wilcoxon rank sum test: p=0.003; r=0.27). The results are illustrated in the categorised histogram/scatter chart (Fig. 3).

Comparing the ICU and HCA, there was no significant association between ward (ICU/HCA) and requirements for ventilatory support, as patients being sent to both wards were all in need of ventilatory support, except for one patient who went to the HCA, and then remained there for only one day before being discharged to the wards

### Length of stay

**Fig. 4:**
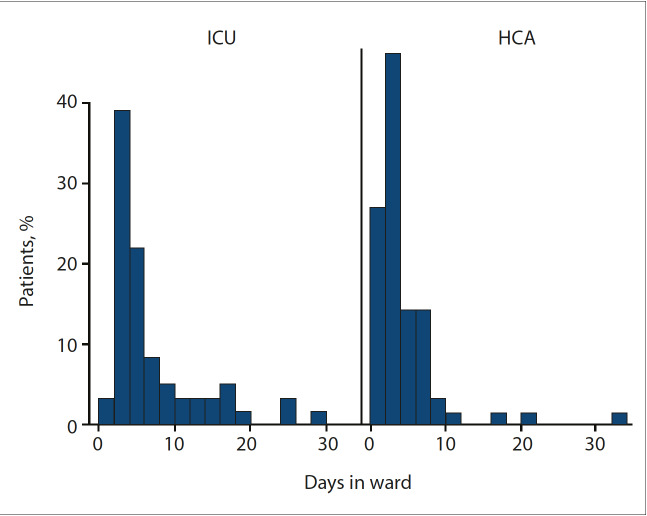
Ward comparison for length of stay. ICU = intensive care unit HCA = high care area

With regard to length of stay, only survivors were analysed (n=122). The median time for length of stay in either the HCA or ICU was three days (IQR 2 - 5; range 1 - 34 days). When directly comparing ICU and HCA, the median number of days in the ward was marginally longer for ICU patients (4 days) than for HCA patients (2 days) (Wilcoxon rank sum test; p<0.0001; r=0.40). The results are illustrated in the categorised histogram/scatter chart (Fig. 4).

### Outcome

Seven patients died, which reflected a mortality rate of 5.4%. Of these, two patients were in the ICU ward (3.3% mortality) and five in the HCA ward (7.4% mortality), which demonstrated no statistically significant association between ward and mortality rate (p=0.45).

When evaluating prognostic scores, the APACHE II score was only calculated in 13 cases, therefore we were unable to make any conclusions with regard to its usefulness.

## Discussion

Worldwide trends have shown that pesticide poisoning, including organophosphate poisoning, is a significant problem in the developing world v. the developed world.^[3,5-7]^ There are very few articles on organophosphate poisoning in sub-Saharan Africa, with those that are available coming either from a significant time ago or covering a paediatric population.^[8,9]^ Our study showed a rapid rise in the incidence of organophosphate poisoning from 2012 to 2014 but a decline in 2015. We feel that this trend, although representative of the situation at the time, could have been influenced by external issues such as HCA/ICU bed and staff availability.

### Demographic profile

Our study showed a male predominance of 68.2%. A review of the literature showed differing results, with a female predominance in some studies, and a male predominance in others.^[10-13,17]^ Reasons given in the literature for these gender differences include farming practices, with the associated occupational exposures; gender-favoured methods of suicide; and gender-specific psychosocial stressors.^[10,13]^ With CHBAH being a predominantly urban hospital, farming practices most likely do not play as great a role as in some of the studies reviewed in the literature, which often covered hospitals in more rural areas. The findings in our study are more probably related to accessibility of the poison, especially in the context of those cases of deliberate self-harm, where impulsivity may result in the use of the most accessible agent in the circumstances, as well as being related to affordability of the poison in an area that comprises mainly lower-income households.

The mean age group affected most by organophosphate poisoning as reviewed in the literature, fell in the 25 - 30-year-old range.^[10-14,17]^ The SA study from the Western Cape (WC) showed that 75% of the cases were under 40 years of age.^[8]^ Our study showed a slightly younger population group with a median age of 30 years, highlighting the age group that is supposedly the most economically active subset of the population. The extrapolated impact of this would not only be felt economically, with breadwinners in the affected families being either temporarily or permanently incapacitated, but would also affect family community structures.

Our study also demonstrated that 85.1% of the patients admitted with organophosphate poisoning had attempted suicide. This figure could be higher, as no reason for ingestion was recorded in 15 cases. This finding is in keeping with previously published research which also found that a significant number of organophosphate admissions were due to suicide attempts.^[6,7,12,13]^ Admissions for organophosphate poisoning are a significant burden on our critical care resources, which are already overburdened by patients admitted for trauma and disease. Our findings also highlight the fact that these substances are easily available. Although the sale of one of the most common organophosphates, aldicarb (Temik), was prohibited under the Fertilizers, Farm Feeds, Agricultural Remedies and Stock Remedies Act (Act 36) of 1947 as gazetted in 2013, and even though the local supplier agreed to halt all sales, as well as collect and dispose of stockpiles of the poison, a version of the substance has become readily available in urban areas.^[19]^ Articles in popular culture literature and social media platforms have blamed the availability of this substance on illegal importation across our borders, which was supported by findings in interviews with the community.^[18]^ Active enforcement of the relevant legislation and control of these substances is desperately needed, with stricter prosecution of those found both in possession as well as those involved in the sale of this poison.

Due to the accumulation of acetylcholine at synapses, patients may present with either muscarinic (increased salivation and lacrimation, urination, defecation, vomiting, miosis, bradycardia and bronchospasm) or nicotinic (muscle weakness and fasciculation leading to paralysis, hypertension, hypoglycaemia, seizures, depression of respiratory and circulatory systems and coma) effects.^[1,2,7]^ A patient exposed to organophosphates may present with varying combinations of the above, with studies showing different frequencies in the presentation of the more commonly encountered signs.^[10,11,14,15]^ In our study, the presence of pinpoint pupils (<1 mm in diameter) was the most common clinical sign, with a GCS score <13 (a cutoff which has been shown previously to be associated with a higher mortality risk)^[20]^ and salivation being the next most common signs, respectively

Whilst indirect laboratory tests of organophosphate poisoning including plasma butyrylcholinesterase (pseudocholinesterase – PCHE) and acetylcholinesterase activity in red blood cells (RCC) are helpful, the diagnosis of organophosphate poisoning is most often made by a positive history of ingestion or exposure, in addition to the classic presentation.^[3,11]^ One of the main difficulties in using these laboratory measurements is that they are not immediately or easily available, and therefore treatment is usually initiated based on clinical diagnosis and/or history alone.^[3,11]^

We found that RCC was taken in only 19.4% of our cases, with a median value of 400 U/L (laboratory reference range 4 752 - 8 225 U/L). Pseudocholinesterase levels were taken in 67.4% of cases, with a median value of 200 U/L (laboratory reference range 4 620 - 11 500 U/L for male and 3 930 - 10 800 U/L for female patients). The literature suggests that depression of the levels of either RCC or PCHE by 20% may indicate significant poisoning, with a decrease of up to 50% occurring in severe cases.^[22]^ With these levels taken into account, the patients in our study demonstrated severe poisoning on the basis of enzymatic inhibition.

Some studies which we examined found an association between mortality, severity and RCC/PCHE levels, with others finding no significant correlation.^[11,22]^ Unfortunately, plasma pseudocholinesterase and RCC were not measured in any of the patients in our study who died. We therefore could not examine this aspect, which may provide an interesting avenue for future research.

Patients in our study had a lower arterial pH (7.16; range 6.74 - 7.47) compared with patients in studies in the literature, with a higher PaCO_2_ (52 mmHg; IQR 40 - 69 mmHg) and a mean HCO_3_ of 19 mmol/L (SD 4.1; range 8.0 - 31.4 mmol/L), indicating our patients were already in respiratory failure on admission, which is in keeping with the high number who required ventilatory support. In the present study, 99.2% of patients admitted to the HCA/ICU required mechanical ventilation, which is much higher than in comparative studies reviewed in the literature, where only 21.2% and 28.6% of patients in the respective trials required mechanical ventilation.^[13,14]^ This high figure may represent selection bias as patients requiring ventilator support are most probably admitted to ICU or HCA at CHBAH, although, as discussed above, many of the patients were already in respiratory failure on arrival. The average length of ventilation for patients in the HCA was 1 day and for ICU 2 days, with a median time on ventilator support of 2 days for both wards. These figures compare favourably with those noted in the literature,^[10,20,23,24]^ although no data were available regarding the duration of ventilation of patients in SA.

The longer the stay in HCA or ICU in these areas, the greater the costs and risk to the patient for further complications, including nosocomial sepsis.^[10]^ Various studies found that the average length of ICU stay for patients who had been exposed to organophosphates was 2 - 10 days, including a study undertaken in the WC, SA in which the average length of ICU stay was 8 days.^[8,10,14,20,23]^ The average length of stay in our study was 3 days. Possible reasons for this difference within SA studies may be related to the fact that the WC study was undertaken 20 years ago, and advances in ICU technology and management, as well as early recognition and initiation of treatment, may play a role. The differences between the compounds used in the poison may also have played a role, with the irreversible binding of the pure organophosphate agents causing a prolonged inhibition of the enzyme as opposed to the reversible carbamate agents which generally result in reversal of symptoms after about 48 hours, depending on the agent and formulation used, mode of consumption and duration of exposure.^[25]^ Our study did not look at the types of organophosphates/ carbamates consumed. There are over 100 known organophosphate agents, and testing would be required for each agent.^[3,25]^ These tests are not easily accessible to a public sector hospital, and would involve unnecessary expense. Our patients’ comorbidities were not taken into account, which could also affect the complications experienced, length of ventilation and therefore length of ward stay.

All our patients were treated with intravenous atropine, with none receiving an oxime, which is due to lack of availability, cost, and conflict in the literature as to potential benefits.^[11,26,27]^ Oximes appear to be effective in only some groups of organophosphates, and are ineffective in poisoning by carbamates.^[26,27]^ Glycopyrrolate has also been used in the treatment of organophosphate poisoning, both independently and in combination with atropine.^[28]^ There are also ongoing studies looking into novel agents for the treatment of organophosphate and carbamate poisoning, the results of which will need to be evaluated in our setting.

Variable mortality rates for organophosphate poisonings are reported in the literature, from as high as above 40% to as low as 2.4%.^[10,20,23,24]^ Reasons suggested for higher mortality rates include the toxic nature of the organophosphates consumed, the lack of readily available antidotes, long distances between hospitals and overstretched staff and resources.^[7,11]^

Our study showed an overall mortality rate of 5.4%, which was generally lower than that found in the literature.^[6,7,10-12,14,24]^ This difference could be related to types of organophosphate poisons consumed, carbamate v. organophosphate poisoning, and more ready access to healthcare in comparison with other developing nations. Many of the studies were conducted on the Indian subcontinent and are from agricultural/rural areas where access to healthcare may be difficult, with inter-hospital transfer of patients to an appropriate facility level possibly affecting outcomes.

We also compared the ICU and HCA with regards to mortality, requirement for mechanical ventilator support, duration of ventilation, and duration of ward stay. There was no significant association between ward and mortality, or indication for ventilation.

Differentiation between patients likely to do well in the ICU/HCA setting, and those with a poor prognosis, by use of a scoring system, has been studied, with several articles examining different scores, particularly in the setting of organophosphate poisoning. Although it is appreciated that outcome and duration of stay are largely influenced by the duration of paralysis and any associated insults such as respiratory arrest or aspiration, a patient’s background comorbidities, age and baseline need to be taken into account. The APACHE II, SOFA (Sepsis-related Organ Failure Assessment), SAPS (Simplified Acute Physiology Score), and GCS all generally show a good correlation with mortality predication, with slight differences in ease of use.^[20,29,30]^ No SA studies evaluating scoring systems in organophosphate poisoning were found. We decided to assess whether the APACHE II score could be used to predict mortality in our setting, as it was already in use in the HCA. Unfortunately, it was recorded for only 13 patients (median value 12, which equates to a 15% mortality rate).^[21]^ We could therefore not comment on its use as a predictor of mortality in our setting.

### Limitations

We recognise that our study has a few inherent limitations, including its retrospective nature, the number of incomplete/lost files and our reliance on non-computed records.

Other limiting factors could include accessibility to beds in HCA/ ICU owing to resource constraints such as staff shortages and ventilator availability. In comparing studies, the type of organophosphate and organophosphate v. carbamate, accessibility to healthcare facilities (especially between rural and urban, low and high socioeconomic status), and sophistication of available healthcare facilities (especially between rural and urban, affluent and poor communities) have not been taken into account.

## Conclusion

Our study provides a better understanding of the demographic profile and clinical presentation of the patients admitted to our ICU/HCA with organophosphate poisoning, as well as a retrospective overview of some of the parameters of the inpatient management of these patients in the wards described. This study will hopefully provide some baseline information for more detailed and focused prospective studies on the typical chemical agents used; comparisons of current treatment options and possible novel agents; acute, sub-acute and long-term complications of organophosphate poisoning; and also more detail to enable focused prevention programmes. In their article on suicide in developing countries, Gunnell and Eddleston^[12]^ proposed that restricting the availability of poisons, improving the safety of pesticides (i.e. by adding an emetic agent to the compound), significant awareness campaigns, and encompassing the public as well as government and health department decision-makers, are steps that should be pursued to limit the effects of pesticide-related suicides in developing countries. Lastly, they suggested that if the previous options are not effective, to ensure that medical management of the repercussions of the toxin ingestion are optimised. These are steps that need to be taken in our setting as a matter of urgency. We hope that our study can provide a base for future studies that will provide more input into this neglected aspect of morbidity and mortality in our healthcare setting.

## References

[1] Balali-Mood M, Abdollahi M (2014). Basic and Clinical Toxicology of Organophosphorus Compounds.

[2] King A, Aaron C (2015). Organophosphate and carbamate poisoning.. Emerg Med Clin North Am.

[3] Osmundson M, Viccellio P (1998). Insecticides and pesticides.. Emergency Toxicology.

[4] Rother HA (2012). Improving poisoning diagnosis and surveillance of street pesticides.. S Afr Med J.

[5] Gunnell D, Eddleston M, Phillips MR, Konradsen F (2007). The global distribution of fatal pesticide self-poisoning: Systematic review.. BMC Public Health.

[6] Mew EJ, Padmanathan P, Konradsen F (2017). The global burden of fatal self-poisoning with pesticides 2006-15: Systematic review.. J Affect Disord.

[7] Eddleston M, Buckley NA, Eyer P, Dawson AH (2008). Management of acute organophosphorus pesticide poisoning.. Lancet.

[8] Bardin PG, van Eeden SF, Joubert JR (1987). Intensive care management of acute organophosphate poisoning. A 7-year experience in the Western Cape.. S Afr Med J.

[9] Balme KH, Roberts JC, Glasstone M (2010). Pesticide poisonings at a tertiary children’s hospital in South Africa: An increasing problem.. Clin Toxicol.

[10] Lee P, Tai DY (2001). Clinical features of patients with acute organophosphate poisoning requiring intensive care.. Intens Care Med.

[11] Eddleston M, Eyer P, Worek F (2005). Differences between organophosphorus insecticides in human self-poisoning: A prospective cohort study.. Lancet.

[12] Gunnell D, Eddleston M (2003). Suicide by intentional ingestion of pesticides: A continuing tragedy in developing countries.. Int J Epidemiol.

[13] Sahin HA, Sahin I, Arabaci F (2003). Sociodemographic factors in organophosphate poisonings: A prospective study.. Hum Exp Toxicol.

[14] Coskun R, Gundogan K, Sezgin GC (2015). A retrospective review of intensive care management of organophosphate insecticide poisoning: Single center experience.. Niger J Clin Pract.

[15] Sungurtekin H, Gurses E, Balci C (2006). Evaluation of several clinical scoring tools in organophosphate poisoned patients.. Clin Toxicol.

[16] Bilgin TE, Camdeviren H, Yapici D (2005). The comparison of the efficacy of scoring systems in organophosphate poisoning.. Toxicol Ind Health.

[17] Razwiedani L, Rautenbach P (2017). Epidemiology of Organophosphate poisoning in the Tshwane District of South Africa.. Environ Health Insights.

[18] Rother H (2010). Falling through the regulatory cracks: Street selling of pesticides and poisoning among urban youth in South Africa.. Int J Occup Environ Health.

[19] (2013). South African Government Gazette: 22 November 2013. http://www.gpwonline.co.za/Search/Pages/Results.aspx?k=%2037037.

[20] Davies JO, Eddleston M, Buckley NA (2008). Predicting outcome in acute organophosphorus poisoning with a poison severity score or the Glasgow coma scale.. QJM.

[21] Knaus WA, Draper EA, Wagner DP, Zimmerman JE (1985). APACHE II: A severity of disease classification system.. Crit Care Med.

[22] Prasad D, Jirli P, Mahesh M, Mamatha S (2013). Relevance of plasma cholinesterase to clinical findings in acute organophosphorous poisoning.. Asia Pac J Med Toxicol.

[23] Sungur M, Güven M (2001). Intensive care management of organophosphate insecticide poisoning.. Crit Care.

[24] Shaikh JM (2008). Management of acute organophosphorus poisoning at a university hospital.. Crit Care.

[25] Roberts JR, Relgart JR (2013). Recognition and Management of Pesticide Poisonings.

[26] Eyer F, Worek F, Eyer P (2009). Obidoxime in acute organophosphate poisoning: 1 - clinical effectiveness.. Clin Toxicol.

[27] Eddleston M, Szinicz L, Eyer P, Buckley N (2002). Oximes in acute organophosphorus pesticide poisoning: A systematic review of clinical trials.. QJM.

[28] Eddleston M, Chowdhury FR (2016). Pharmacological treatment of organophosphorus insecticide poisoning: The old and the (possible) new.. Br J Clin Pharmacol.

[29] Sungurtekin H, Gürses E, Balci C (2006). Evaluation of several clinical scoring tools in organophosphate poisoned patients.. Clin Toxicol.

[30] Bilgin TE, Camdeviren H, Yapici D (2005). The comparison of the efficacy of scoring systems in organophosphate poisoning.. Toxicol Ind Health.

